# Clinical Lectures on Various Forms of Intra-Abdominal Suppuration

**Published:** 1901-12

**Authors:** 


**Clinical Lectures on Various Forms of Intra-Abdominal Suppuration.** By A. H. Tubby. Pp. 101. London: The Medical Publishing Company, Ltd. 1901.

This is a useful little book for students. The statements made in it are clear, and a definite plan of instruction is carefully followed. It will give to the student a very good general idea of the causes, signs, and treatment of intra-abdominal suppuration. The title is, however, rather misleading, for the first 56 out of the 91 pages are devoted to the consideration of appendicitis in its various forms—suppurative and non-suppurative. A better title for the book would have been "Appendicitis and Other Forms of Abdominal Suppuration." We most thoroughly agree with the author that it is impossible to forecast at the beginning of an attack of appendicitis what the outcome will be. There is no more treacherous disease. We do not think Mr. Tubby's statement on page 16, that the contents of an appendicitis abscess always contain a certain amount of fæcal matter, quite accurate. It is, of course, often horribly foul, but not because it contains actual fæces. On the same page it is stated that general peritonitis may be either primary or secondary, and that in the primary form the appendix suddenly sloughs and sets up a peritonitis which is often fatal. But a perforation may set up a peritonitis without any primary localisation of pus; and, moreover, we have more than once found a sloughed appendix in a localised appendicitis abscess. On page 28 we are glad to see perforation of the appendix also given as a cause of general peritonitis, without the formation of any localised abscess. Surely the statement on page 24, which to call attention to its importance is printed in italics, is not carefully expressed. The author says: "In a case of declared appendicitis *the danger is extreme when the temperature is subnormal and the pulse over* 100." In a patient weakened by previous pyrexia, or even by an attack of appendicitis without such pyrexia, the pulse may run up to 100 from excitement, or remain at 100 from general weakness. We think the simple statement of the fact that a normal or subnormal temperature may be present in the most dangerous forms of appendicitis and in general peritonitis, one less likely to mislead the student.

In the consideration of the differential diagnosis of appendicitis we look in vain for guidance as to the diagnosis between a localised appendicitis and a diffuse infection of the peritoneum, and this is often a most anxious matter to the surgeon in charge of the case.

We are glad to see reference in these lectures to the importance of operating early enough and the dangers of delay. Mr. Tubby does not agree with those surgeons who advocate operation in all cases.

